# Type 2 and type 1 diabetes have opposing effects on the systemic murine complement alternative pathway

**DOI:** 10.1016/j.isci.2026.116359

**Published:** 2026-06-12

**Authors:** Lucie Colineau, Olga Kolodziej, Daniel Ajona, Ruben Pio, Anna M. Blom, Ben C. King

**Affiliations:** 1 Division for Protein Chemistry, Department of Translational Medicine, Lund University, Malmö, Sweden; 2 Program in Solid Tumors, Cima Universidad de Navarra, Cancer Center Clínica Universidad de Navarra (CCUN), Pamplona, Spain; 3 Navarra’s Health Research Institute (IdiSNA), Pamplona, Spain; 4 Department of Biochemistry and Genetics, School of Sciences, Universidad de Navarra, Pamplona, Spain; 5 Centro de Investigación Biomédica en Red Cáncer (CIBERONC), Madrid, Spain

**Keywords:** health sciences, biological sciences, molecular biology

## Abstract

Complement factor D (FD) (adipsin), is an adipokine essential for the activation of the complement alternative pathway (AP), contributing to inflammation but also to metabolic homeostasis through C3a production. We investigated the metabolic regulation of *Cfd* expression in mouse models. A diet-induced model of insulin resistance reduced expression of key AP components, including adipose *Cfd*, hepatic *C3*, and factor B. In contrast, two independent type 1 diabetes (T1D) models increased adipose *Cfd* and hepatic *C3* expression, leading to elevated *ex vivo* serum AP activation, and increased serum C3b, indicating enhanced complement activation *in vivo*. Across models, adipose *Cfd* expression showed a strong negative correlation with serum insulin levels, and insulin treatment in T1D mice restored *Cfd* to baseline. These findings reveal opposing regulation of adipose *Cfd* and liver *C3* in type 1 versus type 2 diabetes models, with corresponding effects on systemic complement activity, highlighting a link between insulin, complement regulation, and metabolic disease.

## Highlights

• The adipokine complement factor D (FD) is rate limiting for complement activation
• FD serum levels drop in type 2 diabetic models but expression increases in type 1
• Systemic complement activation increases in type 1 accordingly
• FD negatively correlates with insulin and is normalized by insulin treatment

## Graphical abstract

**Figure:**
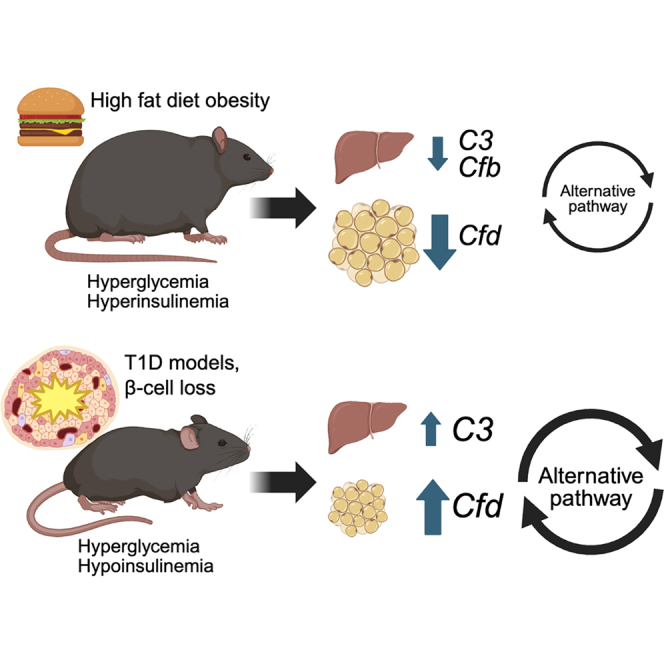

## Introduction

The complement system consists of a network of serum-borne proteins that activate in a proteolytic cascade in response to pathogen- or danger-associated molecular patterns, triggering inflammatory responses.^1^ First discovered over a hundred years ago, complement system has long been seen as a fundamental surveillance and effector mechanism of the innate immune system.^2^ Most complement proteins found in blood are synthesized and secreted from the liver and are acute phase reactants. However, it has recently been discovered that the expression of individual complement components in discrete tissues and individual cells is important for local rather than systemic functions,^3^^,^^4^ and compartmentalization of the activation of complement proteins contributes to cellular and tissue homeostasis.^5^ In particular, there has been a focus on the interplay between complement signaling and immunometabolism, whereby complement activation products signal via specific receptors to reprogram cellular metabolism.^6^ Conversely, it has long been established that whole-organism metabolism can also affect complement gene expression.^7^ While most complement proteins are expressed and secreted from the liver, complement factor D (FD), which is central to the alternative pathway (AP) of complement activation, is produced almost entirely from adipose tissue and was the first identified adipokine, hence its alternative name, “adipsin”. In rodent models of obesity, a marked downregulation of *Cfd*/*adipsin* expression has been observed in adipose tissue.^8^ Consistently, various studies of human cohorts have reported reduced serum FD levels in type 2 diabetes and other metabolic disorders.^9^^,^^10^ Furthermore, serum FD shows a negative correlation with body mass index (BMI), fasting glucose, and HbA1c, while high FD levels are protective against type 2 diabetes.^11^

The AP of complement is continuously and spontaneously activated in biological fluids. This is initiated when complement component 3 (C3), the major central “hub” of the complement system present in serum at concentrations exceeding 1 mg/mL, undergoes spontaneous hydrolysis at its thioester group, forming C3H_2_O at a turnover rate of about 0.4% per hour.^12^^,^^13^ C3H_2_O undergoes a conformational change, allowing serum protein complement factor B (FB) to bind, which is then cleaved by FD. The resultant complex of C3H_2_OBb has been shown to have enzymatic C3 convertase activity and cleaves further copies of C3 to C3b, which undergoes a similar conformational change as C3H_2_O, allowing further FB binding. This self-amplifying process, therefore, consumes C3 and FB but not FD, which is present at the lowest serum concentrations (about 2 μg/mL) and is, therefore, the rate-limiting factor. It should be noted that the effectiveness of C3H_2_O-based convertase in solution is debated,^14^ but alternative models exist, which propose that a conformational change of C3 upon contact with surfaces, including certain lipids, can spontaneously trigger AP activation.^15^ Although the rate of C3 processing by C3H2O- rather than C3b-based AP convertase was shown to be lower, so was its regulation by the complement inhibitor factor H (FH).^16^ Regardless, once C3 cleavage to C3b is triggered by any pathway, including the classical or lectin pathways, the AP acting via FB and FD can then contribute to the majority of subsequent complement activation via the so-called “amplification loop”.^17^ The main inhibitor of this spontaneous self-amplifying activation of the AP is FH, the loss of which in deficient patients or rodent models leads to uncontrolled complement AP activation, complete consumption of C3 and FB from serum, and severe inflammatory pathology as a consequence, which especially targets kidney glomeruli and is thought to be due to a relative absence of other complement inhibitors at this anatomical site.^18^

Spontaneous low-level background activation of the AP leads to the production of the C3 cleavage product, C3a, which is detectable at low levels in serum from healthy individuals. C3a signaling via the C3a receptor (C3aR) has been implicated in several metabolic processes,^19^ including triglyceride metabolism in adipose tissue and the chemotaxis of inflammatory cells to adipose tissue, leading to insulin resistance. Moreover, C3a enhances the secretion of insulin from pancreatic islet beta-cells,^20^ increasing glucose-induced metabolism and ATP production. Based on this, it has been proposed that the AP “tickover” activity augments whole-body metabolism by supporting insulin secretion,^20^^,^^21^ while elevated complement activation can also contribute to inflammation and to insulin resistance in obesity and metabolic disease.^7^^,^^22^^,^^23^^,^^24^^,^^25^ As the rate-limiting factor of the AP, the modulation of FD serum levels by changes in adiposity is, therefore, of direct interest in metabolic disease study. We, therefore, investigated the expression and activity of the AP in rodent models of obesity, insulin-deficient type 1 diabetes (T1D), and fasting and found a significant negative correlation between serum insulin levels and adipose *Cfd* expression.

## Results

### Obesity leads to loss of adipose *Cfd* expression, but also reduced hepatic *C3* and *Cfb*

**Figure 1:**
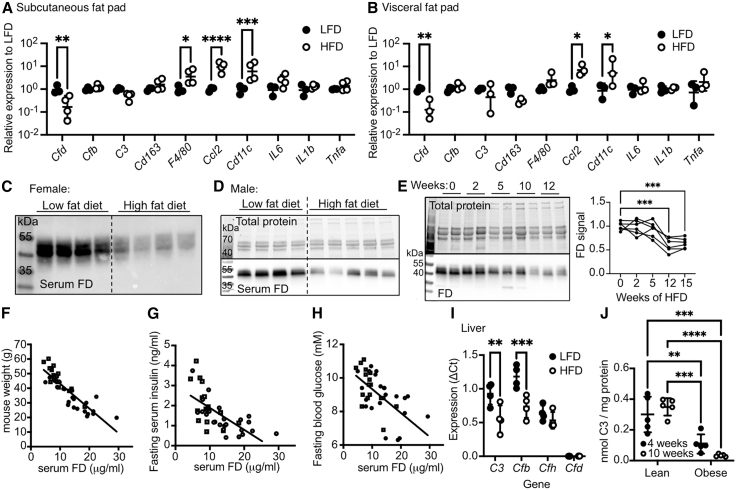
HFD leads to downregulation of the expression of adipose *Cfd* and hepatic *C3* and *Cfb* (A and B) C3-tdTomato reporter mice were placed on LFD or HFD, and expression of inflammatory markers and complement genes were assessed by qPCR in (A) subcutaneous inguinal adipose tissue and (B) visceral epididymal adipose tissue. (C) Western blot for FD in serum from female LFD and HFD mice after 25 weeks of diet. (D) Western blot for FD in serum from male mice after 15 weeks of diet. (E) Left: representative western blot from sequential serum samples from 2 male mice on HFD. Right: densitometry for serum FD signal in western blots of 6 male mice over time. (F–H) Serum FD levels, measured by ELISA, plotted against individual mouse body weight (F), fasting insulin (G), and fasting blood glucose levels (H). (I) Expression of main alternative pathway components in livers of LFD and HFD mice. (J) Protein levels of C3 in liver homogenates from lean control or ob/ob mice on the C57Bl/6 background. WAT, white adipose tissue. In (A)–(C), there were 4 mice per group, except for HFD visceral fat pad, which had 3 samples. In (F)–(H), measurements are from a total of 42 individual mouse samples. For (I) and (J), there were 4–5 mice per group. Data represent mean ± SD. ∗*p* < 0.05, ∗∗*p* < 0.01, ∗∗∗*p* < 0.001, ∗∗∗∗*p* < 0.0001, as tested by two-way ANOVA. See also Figure S1.

We first compared the expression of inflammatory markers by qPCR in both subcutaneous and visceral fat from female C3-TdT reporter mice^26^ placed on high-fat diet (HFD) or matched low-fat diet (LFD) for 28 weeks (Figures 1A and 1B). Here, we found that in both fat pads, expression of CCL2 (also called monocyte chemoattractant protein 1, MCP1) and inflammatory macrophage markers were increased in HFD, consistent with previous findings.^27^^,^^28^ We also found that *Cfd* expression was significantly downregulated, roughly 10-fold. Adipose tissue expression of *C3* and *Cfb* were negligible but unchanged. There were similar levels of gene expression in visceral as in subcutaneous fat. The downregulation of *Cfd* expression was reflected by a stark reduction of FD protein levels in circulating serum (Figure 1C). A similar downregulation of FD protein was seen in serum of male mice on HFD (Figure 1D), with levels decreasing over time (Figure 1E). We also consulted available data from total mRNA microarrays from adipose tissues of LFD/HFD wild-type (WT) male mice on both C57Bl/6 and 129S6/SvEvTac backgrounds,^29^ and in both cases, *Cfd* was among the strongest and most significantly downregulated transcripts (Figures S1A and S1B).

We also measured FD by ELISA in serum samples from multiple strains of mice, including WT, C3-KO, as well as floxed-C3 reporter mice and beta-cell specific C3-KO mice, which had been on LFD or HFD, and found that FD levels had an overall strong negative correlation with mouse total body weight and both fasting blood insulin and glucose levels (Figures 1F–1H).

While FD is the rate-limiting factor for the AP, C3 and CFB also make up the convertase while FH negatively regulates it.^30^ These serum factors are produced overwhelmingly by the liver. On assessing their expression in liver of our female HFD-fed mice compared with LFD controls, we saw significant downregulation of *C3* and *Cfb* expression but no change in *Cfh* mRNA levels (Figure 1I). Analyzing publicly available RNA microarray data^31^ from male mice of the genetic hyperphagic ob/ob mouse model on a C57Bl/6 background, we also observed similar significant downregulation of hepatic *C3* and *Cfb* expression in obese compared to lean control mice (Figures S1C and S1D). This downregulation was not global; *C3* expression was significantly increased in isolated islets of the obese mice (Figure S1E), as we have also demonstrated for other diabetic models.^32^ In this previous study, liver metabolites and protein were also quantified. We extracted data for C3 protein, which was also significantly decreased in liver lysates of obese compared with lean male mice (Figure 1J).

### Streptozotocin-induced T1D results in increased complement AP gene expression

**Figure 2:**
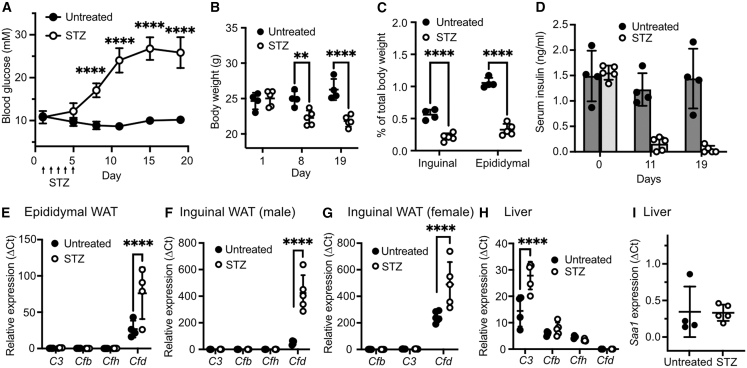
Multiple low-dose streptozotocin-induced T1D results in weight loss and *Cfd* upregulation (A) Blood glucose levels in control mice (*n* = 4) and STZ-treated mice (*n* = 5) over time. (B) Total body weight in control and STZ-treated mice. (C) Inguinal and epididymal fat pad weights in the two groups of mice at the experimental endpoint. (D) Serum insulin levels in control and STZ-treated mice. (E and F) Gene expression in visceral (epididymal) (E) and subcutaneous (inguinal) (F) fat pads of control and STZ-treated male mice at endpoint. (G) Gene expression in the subcutaneous fat pad of female mice with or without equivalent STZ treatment. (H) Gene expression in livers of control and STZ-treated male mice. (I) Expression of inflammatory marker gene *Saa-1* in livers of control and STZ-treated male mice. For all images, *n* = 4 untreated mice and 5 STZ-treated mice except for (E), where one STZ sample was excluded for poor extracted RNA quality; for (G), *n* = 5 for both STZ-treated and untreated mice. Data represent mean ± SD. ∗∗*p* < 0.01 and ∗∗∗∗*p* < 0.0001, as tested by two-way ANOVA. See also Figure S2.

In contrast to animal models of obesity-related diabetes, we next asked what happened to FD levels in animal models of T1D. Here, a loss of beta-cell mass leads to an almost total lack of insulin, preventing glucose utilization and resulting in weight loss. To investigate the effect of this on FD production, we utilized a multiple low-dose streptozotocin (STZ) model, which induces direct beta-cell apoptosis but also release of islet autoantigens, infiltration of inflammatory cells into islets, and T cell mediated beta-cell destruction.^33^ Within 10 days of STZ induction, male WT C57Bl/6N mice became diabetic, with increased blood glucose (Figure 2A), reduction in total body mass as measured at days 8 and 19 (Figure 2B), and significant loss in proportional mass of both inguinal (subcutaneous) and epididymal (visceral) fat pads (Figure 2C). Measurement of serum components by ELISA confirmed near total loss of insulin production (Figure 2D). Surprisingly, in contrast to obese mouse models, STZ-induced diabetic mice had significant and strong upregulation of *Cfd* expression in visceral (Figure 2E) and especially, subcutaneous (Figure 2F) fat pads. This significant upregulation of *Cfd* in STZ-treated male mouse subcutaneous fat was also replicated in STZ-treated female mice (Figure 2G). Other AP components had only negligible expression in adipose tissue. In comparison with and in contrast to obese mice, the liver of male mice showed significant upregulation of *C3*, with no significant changes in *Cfb* or *Cfh*, and negligible expression of *Cfd* (Figure 2H). We also found a significant upregulation of hepatic *C3* expression in publicly available RNA array data of an independent cohort of male STZ mice^34^ compared with untreated controls (Figure S2A). Many complement components are acute phase proteins and can be upregulated in response to inflammation, but we found no change in hepatic expression of serum amyloid A (SAA-1) (Figure 2I), an acute phase reactant that is a sensitive murine inflammatory marker.

**Figure 3:**
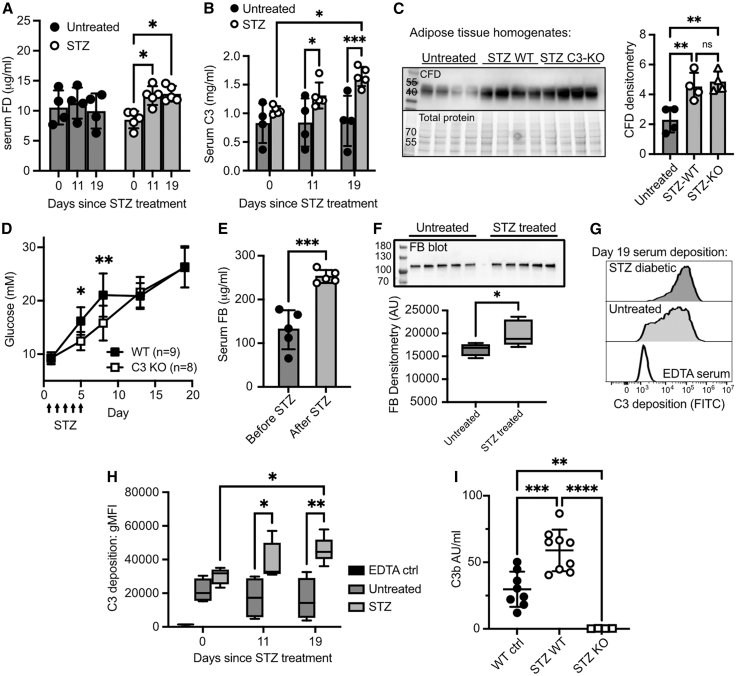
Expression changes in STZ-induced diabetes leads to increased complement in serum and increased alternative pathway activation (A) Serum levels of FD in untreated and STZ-treated male mice. (B) Serum C3 levels in untreated controls and STZ-treated male mice. (C) Western blot for FD in homogenates of inguinal adipose tissue from control mice and STZ-treated WT and C3-KO mice. Densitometry quantification shown on the right. (D) Blood glucose levels in STZ-treated WT or C3-KO male mice over time. (E) Serum FB levels in STZ-treated male mice before and after treatment. (F) Western blot for FB in serum samples of untreated and STZ-treated male mice (top), and quantification by densitometry (bottom). (G) Example flow cytometry histograms of C3 staining of zymosan beads after incubation with serum from untreated or STZ-treated cage-mate mice in EGTA buffer, allowing only AP activation. EDTA completely inhibits complement and acts as a negative control. (H) Results of C3 deposition onto zymosan beads from serum taken at different time points from untreated or STZ-treated male mice. (I) Serum C3b levels in STZ-treated or mock-treated male mice and in STZ-treated C3-KO controls, as measured by ELISA. For all images, *n* = 4 untreated mice and 5 STZ-treated mice, as in Figure 2, except for (C), with groups of 4; (D), with groups as stated in the figure; and (I), with *n* = 8, 9, and 4 for untreated, STZ-treated, and STZ-treated KO groups, respectively. Data represent mean ± SD. ∗*p* < 0.05, ∗∗*p* < 0.01, ∗∗∗*p* < 0.001, ∗∗∗∗*p* < 0.0001, as tested by two-way ANOVA, *t* test (E), or one-way ANOVA (C and I).

To investigate whether increased mRNA levels also resulted in increased amounts of proteins in circulation, we tested serum samples by ELISA. STZ-treated diabetic mice also had increased serum levels of both FD and C3 proteins (Figures 3A and 3B). Protein levels of FD were also clearly increased at the site of expression, in homogenates of subcutaneous adipose tissue (Figure 3C), and this was also true in C3-KO mice, showing that complement activation was not required for *Cfd* upregulation. It has been reported that C3 is required for STZ-induced diabetes induction,^35^ although this was not confirmed in a separate study.^36^ We found that C3-knockout (KO) and WT mice reached identical levels of blood glucose but with a slightly slower rate of induction in KO, with significantly lower blood glucose levels at earlier time points (Figure 3D). C3 was, therefore, not required for the induction of diabetes, although it accelerated progression.

We hypothesized that the increase in serum levels of AP components FD and C3 in STZ-induced WT diabetic mice would increase turnover and therefore, consumption of FB, which was unchanged at mRNA level in the liver. However, serum levels of FB were also increased at the protein level (Figure 3E), as was also confirmed by western blot (Figure 3F), possibly suggesting an increase in extra-hepatic production.

In order to verify whether the increases in serum C3, FD, and FB led to increased AP potential, sera from STZ-treated and control mice were incubated with zymosan particles in the presence of EGTA, and C3 deposition was measured by flow cytometry (Figures 3G and 3H). Serum from STZ-treated diabetic mice potentiated higher levels of C3 deposition than untreated littermate controls, confirming higher AP complement activity. Finally, to confirm our hypothesis that increased levels of complement AP components, and therefore, increased AP activation potential leads to increased tick-over, we measured complement activation product C3b in mouse serum. STZ-induced diabetic mice had significantly higher levels of C3b in blood serum, while a lack of signal from C3-KO mice verified the assay specificity (Figure 3I).

### Spontaneously diabetic lean Akita mice also demonstrate complement upregulation

**Figure 4:**
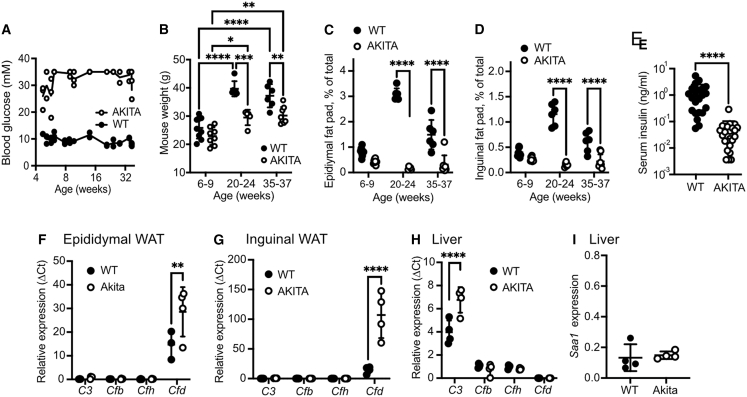
The Akita mouse model of T1D also leads to upregulation of complement alternative pathway gene expression (A) Blood glucose levels in Akita (*n* = 24) mice and WT (*n* = 23) littermates of a range of ages. (B) Body weight of Akita and control mice at various ages. (C and D) Visceral epididymal fat pad (C) and subcutaneous inguinal fat pad (D) weights at age of euthanisation. (E) Serum insulin levels in Akita mice and WT littermates. (F and G) Complement AP gene expression in visceral (epididymal) fat pad (F) and subcutaneous inguinal fat pads(G). (H) Complement AP gene expression in livers of Akita and WT littermates. (I) *Saa-1* gene expression in the livers of the same mice. Each symbol represents the mean values from one mouse. For (F)–(I), qPCR was performed on samples from age-matched pairs of 18- to 24-week-old mice, with a total of 4 per group (WT, Akita). Data represent mean ± SD. ∗*p* < 0.05, ∗∗*p* < 0.01, ∗∗∗*p* < 0.001, ∗∗∗∗*p* < 0.0001, as tested by two-way ANOVA or *t* test (E).

STZ injection can potentially have off-target toxic effects on tissues such as the liver.^37^ We, therefore, also investigated a second, spontaneous model of rodent T1D. Akita mice contain a mutation in the insulin-2 gene, causing protein misfolding, endoplasmic reticulum (ER) stress, and apoptosis induction in beta-cells, with diabetes occurring at an early age.^38^^,^^39^ Compared with WT littermates, Akita mice developed high blood glucose from 4 to 5 weeks after birth (Figure 4A). Consistent with the T1D phenotype, Akita mice put on less weight with time (Figure 4B) and had much smaller visceral and subcutaneous fat pads (Figures 4C and 4D). The loss of insulin production was also confirmed by ELISA (Figure 4E). Consistent with the STZ model, *Cfd* was strongly upregulated in both visceral (Figure 4F) and subcutaneous fat (Figure 4G), with highest changes in subcutaneous fat. Also consistent with the STZ T1D model, *C3* was significantly upregulated in liver, with no significant changes in *Cfb* or *Cfh* expression (Figure 4H), and there was an absence of changes in the expression of the acute phase inflammatory marker SAA (Figure 4I).

**Figure 5:**
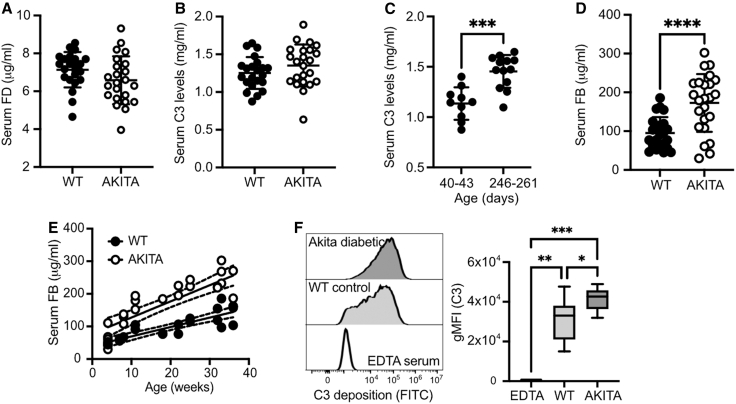
T1D Akita mice have increased serum FB levels and alternative pathway activation (A) Serum FD levels in Akita and WT littermates, as measured by ELISA. (B) Serum C3 levels in the same mice. (C) Serum C3 levels in “young” versus “older” mice. (D) Serum FB levels in all Akita and WT littermate mice. (E) Serum FB levels in the same mice, stratified over time, plotting line of best fit and 95% confidence intervals. (F) C3 alternative pathway deposition onto zymosan beads from age-matched Akita or WT littermate mice. Left: example histograms from cage-mate mice; right: quantification of C3 deposition results from serum taken from age-matched pairs of mice aged from 18 to 24 weeks (*n* = 5 per genotype). Each data point represents mean value from an individual mouse. Data represent mean ± SD, with ∗*p* < 0.05, ∗∗*p* < 0.01, ∗∗∗*p* < 0.001, ∗∗∗∗*p* < 0.0001, as tested by one-way ANOVA (F) or *t* test (C and D). In (E), lines show best fit and 95% confidence intervals.

Despite the dramatic loss in adipose tissue mass, serum FD protein levels were maintained in Akita mice, as measured by ELISA (Figure 5A), while there was no significant difference in serum C3 levels (Figure 5B). Interestingly, the Akita cohort showed an increased serum C3 level with age, regardless of genotype (Figure 5C). We also assessed serum FB levels and found that these were not only increased in Akita mice (Figure 5D) but they also increased with age (Figure 5E). Assessment of sera from age-matched Akita mice and littermate controls also showed significantly increased AP activity in serum samples from the diabetic mice compared with healthy cage-mates (Figure 5F).

### Weight loss alone does not alter *Cfd* expression levels in adipose tissue

**Figure 6:**
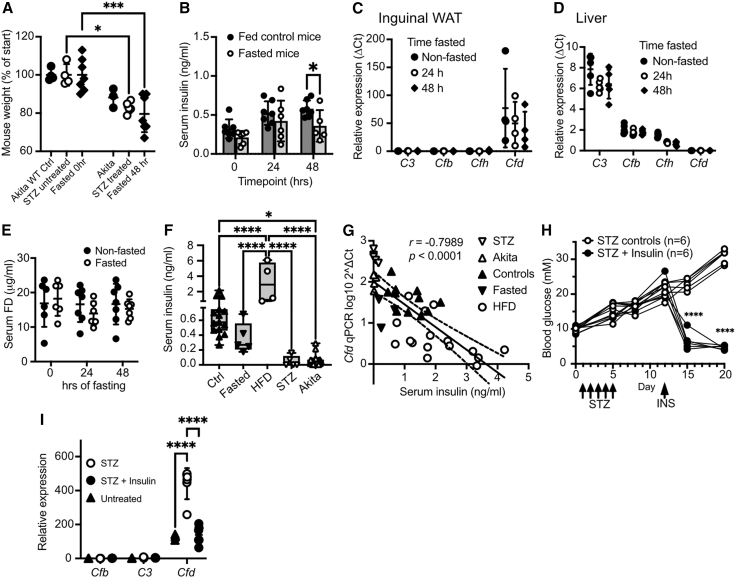
Fasting-induced weight loss alone does not alter complement gene expression (A) Comparison of relative total weight loss in the fasting model compared with the Akita and STZ-induced diabetes T1D models, comparing age-matched mice only. (B) Serum insulin levels in fasted mice and non-fasted controls. (C) Complement AP gene expression in adipose tissue of fasted mice. (D) Complement AP gene expression in livers of fasted mice. (E) Serum FD levels from fasted and non-fasted mice at various time points. (F) Comparative serum insulin levels from different mouse groups used in the study. (G) Correlation between subcutaneous white adipose tissue *Cfd* expression and serum insulin levels in comparable age-matched mice under different treatments. (H) Blood glucose levels in the male cohort of STZ-treated mice, including treatment with insulin implants. (I) Complement AP component expression in subcutaneous inguinal fat pads from mice in (H) as well as matched male untreated control mice (*n* = 4). Each symbol represents the mean values from one mouse. Data represent mean ± SD, except in (G), where lines show best fit and 95% confidence intervals. ∗*p* < 0.05, ∗∗∗*p* < 0.001, ∗∗∗∗*p* < 0.0001, as tested by two-way ANOVA or one-way ANOVA (F).

Although the obese HFD mice and both T1D models had increased blood glucose levels, they had opposing changes in adipose *Cfd* expression. One factor that correlated consistently with FD levels in all mice was body weight, with increased adipose mass being associated with a loss in adipose *Cfd* expression, and decreased adipose tissue mass in T1D models being associated with a compensatory increase in *Cfd* expression. To investigate a direct link between weight loss and *Cfd* expression, naive mice were subjected to a period of 48 h of short-term starvation (STS). This induced a dramatic weight loss over 48 h (Figure 6A), equivalent to or greater than the relative weight loss seen in T1D models. There was only a significant change in basal insulin levels in serum from mice fasted for 4 h (Figure 6B). Despite weight loss, fasting for up to 48 h did not lead to significant changes in mRNA expression of *Cfd* in subcutaneous adipose tissue (Figure 6C) nor in *C3* expression in liver tissue (Figure 6D). No changes in serum FD levels were seen by ELISA (Figure 6E), confirming that weight loss alone did not alter FD levels.

Taking all the mouse models together, *Cfd* expression, therefore, did not consistently correlate with blood glucose level or correlate directly with body weight. We, therefore, tested the correlation of serum insulin levels (Figure 6F) with adipose tissue *Cfd* expression in all comparable age-matched mice used and found a significant negative correlation that was consistent across all experimental models (Figure 6G). To verify the upstream effect of insulin on *Cfd* expression, we conducted a new experiment of STZ-induced diabetes in male mice. After 1 week of diabetes onset, we treated half the mice with subcutaneous implants that slowly release insulin. This rapidly normalized blood glucose levels (Figure 6H). After one more week, we isolated subcutaneous adipose tissue and tested for AP component expression. We found that in insulin-treated mice, *Cfd* had returned to expression levels found in untreated control mice (Figure 6I), confirming the upstream effect of insulin on expression of *Cfd*, the rate-limiting factor of the complement AP.

## Discussion

We have discovered opposing effects in mouse models of obesity-related type 2 diabetes and insulin deficient T1D for the systemic complement AP, with not only the loss of *Cfd* expression in adipose tissue in obese mice but also loss of hepatic *C3* expression. In contrast, in T1D models involving beta-cell destruction accompanied by hypoinsulinemia and weight loss, not only was there a compensatory strong upregulation of *Cfd* expression in adipose tissues but also significant increases in hepatic *C3* expression, with overall result of increased AP activation potential in serum, as evidenced by increased C3 deposition in *ex vivo* assays and increased serum levels of complement activation product C3b. Considering recent work showing crosstalk between the spontaneous activation of the AP and metabolic functions of pancreatic islets as well as adipose tissues, this has implications not only for metabolic homeostasis, but also for the potential involvement of complement in the pathology of diabetic complications.

It has long since been established that rodent models of obesity lead to loss of *Cfd* expression in adipose tissue,^40^ the main source of this adipokine. However, to our knowledge, a similar downregulation of hepatic expression of the central complement component C3 has not been described in animal models of obesity until now, likely also contributing to the loss of serum complement activity previously described in these models. Although C3 mRNA expression was downregulated in our obese mice, we did not find a significant change in serum C3 levels. Serum levels of complement proteins are a balance between expression and consumption. With decreased amounts of serum FD in obese mice, the spontaneous activation of the AP will be reduced, and therefore, C3 can be expected to have a longer half-life in serum. Indeed, in *Cfd*-knockout mice, spontaneously hydrolyzed C3 is not cleared from serum but accumulates, leaving higher C3 levels compared with WT mice.^41^ Similarly, serum levels of FB are also higher in otherwise healthy FD-KOs compared with WT mice,^41^ providing evidence for spontaneous consumption of AP components in serum. We also see elevated levels of FB in the serum of our C3-KO mice, again suggesting continuous background levels of consumption of FB by the AP in normal serum, which would lead to low level production of C3a. The decreased levels of FD in obese models, therefore, lowers the potential for AP activation, as previously described,^41^ and this decrease of consumption of AP components may result in the unchanged levels of serum C3 despite decreased hepatic production, especially in context of unchanged expression of *Cfh*, the main inhibitor of the fluid phase AP.

In T1D models, characterized by significant weight loss and reduction of fat pad mass, we also saw strong upregulation of adipose *Cfd* expression and increases in hepatic *C3* expression. In STZ-treated mice, serum levels of both C3 and FD were increased, consistent with increased mRNA transcripts. Interestingly, serum FB levels were also increased despite no significant change in hepatic transcripts, suggesting a possible increase in extra-hepatic production or altered regulation at a post-transcriptional level. Consistent with this, serum FB levels were also increased in Akita diabetic mice compared with controls, with no significant change in hepatic mRNA levels. Both of these models, therefore, suggest an extra-hepatic source of FB. It has been found that hyperglycemia upregulates Cfb expression in mouse kidneys, and that CFB is upregulated in podocytes from humans with diabetic kidney disease.^42^ In addition, targeting FB alleviated both AP activation and kidney disease in these diabetic mice.^42^ In Akita mice, there was no significant increase in serum C3 levels, possibly due to increased activation and consumption, and also no significant increase in measured serum FD levels. This could be due to two factors. Firstly, although *Cfd* expression in adipose tissue was highly upregulated in these mice, the loss of adipose tissue mass was also the greatest. Secondly, FD is a small serum protein of about 25 kDa, although further glycosylated in mice, and is filtered through the kidney glomeruli. Akita mice have especially high blood glucose levels and have a higher rate of polyuria,^43^ and a higher rate of loss of serum FD levels via the kidneys can, therefore, be expected. Similar as aforementioned, serum C3 levels in HFD mice were also not significantly reduced despite a reduction in hepatic mRNA levels. This is due to the reduction of serum FD levels that occurs in obesity, which has been previously demonstrated to lead to a reduction in serum AP turnover and therefore, C3 consumption,^8^^,^^20^^,^^21^ and to limit AP activation—exactly the opposite effect as to what we now also see in T1D mice with elevated serum FD.

Our findings in mouse models are also supported by human data. It has been observed that serum FD levels are reduced in patients with type 2 diabetes,^9^^,^^10^ and that they are negatively correlated with BMI, HbA1c, and fasting blood glucose in cohorts of healthy and newly diagnosed type 2 diabetic patients,^11^ although this has not been replicated in all studies.^44^ It has also been found that short-term HFD feeding leads to down regulation of serum C3 in healthy males.^45^ No significant increase in serum FD levels was seen in human patients with anorexia nervosa who had low BMI,^46^ consistent with the normal levels seen in our fasted weight-loss model mice. However, there is little available data from untreated human patients with T1D, probably due to the importance attributed to rapidly initiating insulin therapy to regulate blood glucose homeostasis. Our results show that successful insulin treatment of STZ-induced T1D mice restores *Cfd* expression to levels seen in healthy mice, and this implies that when properly managed, T1D patients may also have normal complement AP activity.

HFD is associated with systemic low-grade chronic inflammation,^7^ as also shown by the upregulation of infiltrating macrophage markers that we saw in adipose tissues. This could be a potential reason for adipose tissue dysfunction and loss of *Cfd* expression. We did not, however, see similar inflammation in the T1D models, as shown by a lack of SAA-1 upregulation in the liver. We also did not see upregulation of inflammatory markers tested in adipose tissues (not shown). While STZ induction may induce a *de novo* autoimmune anti-insulin response,^35^ the Akita mouse is noted for its lack of islet inflammation and insulinitis,^47^ therefore lacking an inflammatory component. We, therefore, rule out inflammation as the stimulus that drives changes in AP component expression in our T1D models.

FD replenishment in obesity-induced deficiency improved insulin secretion and normalized blood glucose levels in obese mice,^20^ proposed to be due to the AP production of C3a; C3a augments insulin secretion from islets by boosting beta-cell metabolism.^20^ In human cohorts, serum FD levels were also positively correlated with first-phase insulin secretion.^9^

C3a production and C3aR signaling are also proposed to prevent islet atrophy and support beta-cell function and development.^21^^,^^48^ In adipocytes, C3a and C5a signaling have insulin-like effects, and use of receptor antagonists inhibit weight gain and tissue inflammation in diet-induced obesity models.^49^ Studies of C3aR/C5aR KO mice also show attenuated diet-induced obesity,^23^^,^^50^ and C3aR also regulates adipose tissue thermogenesis in a sex-dependent manner.^51^ This raises questions as to the site of complement activation and C3a production. Altered systemic levels of complement proteins could influence activation potential locally in tissues. Early studies showed that isolated adipose tissue was capable of secreting all components required for AP activation, and that this results in C3a production in an autocrine manner.^52^ Another study found that C3a production peaked post-prandially in human adipose tissue, as detected in draining vasculature, which was not reflected in circulating arterial blood.^53^ We also found evidence for autocrine AP activation from isolated human islets, with secreted FB being cleaved to Bb in supernatants.^54^ This local activation at the proposed site of action would be necessary due to the rapid inactivation of C3a by serum peptidases. The measured systemic changes that we find in this paper are reflective of altered local production, such as the increase in local and also systemic levels of FD in both adipose tissue and serum in STZ-treated mice (Figure 3). Further work is needed to investigate the local homeostatic production of C3a in tissues *in vivo* and how this could be influenced by the local and global alterations of AP components that we describe in this paper.

Complement system may also modulate the development of T1D via its role in the mounting of autoimmune adaptive responses.^35^ In this study, C3-KO mice still became diabetic after STZ administration, but the development of diabetes was significantly reduced at earlier time points, implicating a role of the complement system. This is also supported by gain-of-function polymorphisms in C3 that are associated with increased risk of T1D in human DR4/4 patients.^55^ Further investigation would be required to conclusively link the AP to this process.

A single-cell atlas of mouse adipose tissues shows that in both visceral and subcutaneous tissue, *Cfd* is expressed most strongly by a specific sub-population of adipocytes; this population is reduced in number under HFD, and their expression of *Cfd* is also downregulated.^56^ This population also expressed high levels of genes involved in triglyceride metabolism, such as *Gpx1* and *Scd1*. However, whether this population increases in proportion under T1D conditions was not studied. FD secretion from adipocytes is also downregulated by hypoxia,^57^ and hypoxic conditions arise in murine obese adipose tissue,^58^ although conflicting results have been presented in human studies.^59^ In addition, FD expression is strongly linked to adipocyte differentiation, and evidence of de-differentiation of adipose populations has been revealed in obesity, with many genes involved in adipogenesis downregulated in adipose tissue in obesity and diabetes.^60^ In contrast, plasma from patients with T1D was shown to enhance adipocyte differentiation *in vitro*.^61^ More work is required to identify changes to adipocyte subpopulations within T1D. Further work is also required to verify the effect of insulin treatment on reversing the effects seen in T1D mice, as well as to assess the implications of altered AP potential and activation in peripheral tissues in type 1 and type 2 diabetes, both in homeostatic processes of adipose tissues, pancreatic islets, and other organs but also in potential pathological roles of complement proteins in diabetic complications.^7^

### Limitations of the study

Limitations of this study include small group sizes used to limit animal use, although we have used multiple models and both male and female groups. Potential off-target effects of STZ treatment, for example, hepatic toxicity, were mitigated by our use of the additional Akita model. Serum measurements of complement proteins integrate both expression but also consumption of complement proteins during ongoing complement activation; we mostly inferred AP potential and activity by *ex vivo* activation assays, although direct measurement of serum C3b supported our hypothesis. This report forms a basis for understanding the differential effects of type 1 and type 2 diabetes on basal complement AP activation potential in animal models for studying these phenomena.

## Resource availability

### Lead contact

Requests for further information and resources should be directed to and will be fulfilled by the lead contact, Anna M Blom (anna.blom@med.lu.se).

### Materials availability

This study did not generate new unique reagents.

### Data and code availability

This paper analyzes existing, publicly available data, accessible as described in the key resources table. Raw data generated in the paper are available on request from the lead contact.

## Acknowledgments

The study was supported by the 10.13039/501100012529Diabetes Wellness Sverige, the Crafoord Foundation, the Åke Wibergs Foundation, the 10.13039/501100006285Magnus Bergvall's Foundation, the 10.13039/501100006189Albert Påhlsson Foundation, the 10.13039/501100004063Knut and Alice Wallenberg Foundation, the 10.13039/501100008550Diabetesfonden, 10.13039/501100004359Swedish Research Council, Strategic Research Area Exodiab, Dnr 2009-1039, the Swedish Foundation for Strategic Research (LUDC-IRC; grant agreement number IRC15-0067), 10.13039/501100004587Instituto de Salud Carlos III (PI23/00573 and AC24/00153), co-funded by the 10.13039/501100000780European Union and 10.13039/501100016120Departamento de Salud, Gobierno de Navarra, and cofunded at 50% by 10.13039/501100008530Fondo Europeo de Desarrollo Regional 2014-2020 (51-2021). The authors would like to thank Marina Mckay, Julia Slaby, and Lukasz Lichon for technical assistance, and Professor Maria Gomez for providing Akita mice. Olga Kolodziej, Julia Slaby, and Lukasz Lichon were students of University of Rzeszów, Poland. Graphical abstract created in BioRender by King, B. (2025): https://BioRender.com/379vpfn. Grant providers were not involved in the study design.

## Author contributions

L.C. and B.C.K. conceptualized the study; L.C., O.K., D.A., and B.C.K. investigated and collected data; D.A. and R.P. contributed resources; B.C.K. and A.M.B. supervised the study; B.C.K. wrote the original draft of the paper; B.C.K., A.M.B., and R.P. acquired funds; all authors reviewed and edited the manuscript.

## Declaration of interests

The authors declare no competing interests.

## STAR★Methods

### Key resources table

**Table:** 

REAGENT or RESOURCE	SOURCE	IDENTIFIER
**Antibodies**		

Sheep anti-mouse FD	R&D Systems	Cat #AF5430; RRID:AB_1655868
Goat anti-mouse FB	Complement Technologies	Cat #A235
Rabbit anti-C3	AbCam	Cat #ab200999; RRID:AB_2924273
Goat anti-mouse C3	ICN	Cat #55500

**Critical commercial assays**		

mouse factor B ELISA	Novus Biologicals	Cat #NBP2-75243
mouse factor D/adipsin DuoSet	R&D Systems	Cat #DY5430
mouse total C3 ELISA kit	Abcam	Cat #ab263884; RRID: AB_3075457
mouse C3b ELISA kit	Hycult	Cat #HK216

**Deposited data**		

Microarray data from low-fat diet (LFD) and high-fat diet (HFD)-fed male mouse epididymal fat pads	https://www.ncbi.nlm.nih.gov/geo/geo2r/?acc=GSE123394	GSE123394
Array data from male C57Bl/6 lean and ob/ob mice	Attie lab diabetes database	http://diabetes.wisc.edu/
Array data from livers of STZ-treated mice and controls	https://www.ncbi.nlm.nih.gov/geo/query/acc.cgi?acc=GSE39752	GSE39752

**Experimental models: Organisms/strains**		

Mouse, Akita	Maria Gomez	C57BL/6-*Ins2*^*Akita*^
Mouse	Own breeding	C57BL/6N

### Experimental model and study participant details

#### Animals

All mice were housed in specific pathogen-free conditions at controlled ambient temperatures in individually air-conditioned cages with 12 hour light/dark cycles, with environmental enrichment of soft bedding, chew sticks, and cardboard rolls, and daily observation. Humane endpoints included over 20% loss of body weight and blood glucose levels over 30 mM. To study the effects of diet-induced obesity, from 8 weeks of age, C57Bl/6N mice from our own breeding were placed on high-fat diet with 60% of calories from fat (Research Diets, #D12450Ji), or on control low-fat diet (Research Diets, #D12492i) for 6 months, before tissues were harvested. Animal placement into control diet or HFD cages was random. To assess possible effects of sex, groups of male mice were also placed on high fat diet, from 8 weeks of age, as described in the text. For the multiple low-dose streptozotocin-induced type 1 diabetes model, male C57Bl/6N mice (n = 5) at 8 weeks of age were injected intraperitoneally with 50 mg/kg streptozotocin (Merck Millipore), every day for 5 days. Streptozotocin is unstable once in solution; to mitigate this confounder, solutions were prepared fresh immediately before injection, and the order of mice to be injected was rotated each day. Untreated age-matched cage-mate littermates were used as controls (n = 4). STZ-treated mice were chosen by random per cage (total of 2 cages) based on arbitrary numbering of mouse identities, so that treated and untreated mice were co-housed, to prevent cage-specific effects. Tissue samples were taken at the experimental endpoint 2 weeks later. Another experiment was carried out on age-matched groups of 8-10 week old male mice to test the effect of insulin therapy. The experiment was also carried out in age-matched 10-14 week old male C3-WT or KO mice on the same C57Bl/6N background to address the influence of complement. The effect of STZ was also assessed in female C57Bl/6N mice to address sex-specific effects; female mice are more resistant to streptozotocin and so a dose of 60 mg/kg was used.

For a spontaneous T1D model, male Akita mice, which develop spontaneous diabetes at a young age due to beta-cell destruction,^38^^,^^39^ were provided by Professor Maria Gomez (Lund University) (n = 24 Akita mice and 23 WT littermates, between 5 and 34 weeks of age). Age-matched animals at the mid-range of the colony were chosen for tissue RNA extraction, to represent the colony average. Spontaneous diabetes is far less penetrant in female Akita mice and so females were not assessed in this model.

For the fasting model of weight loss, groups of female C57Bl/6 mice (n = 7 per group, 8 weeks of age) were subjected to food removal for 24 or 48 h, with a control group allowed unrestricted access to food. Tissue samples were taken at the endpoint, and serum samples at the experimental start point and every 24 h. For *in vivo* short-term starvation (STS), C57Bl/6J mice underwent complete food deprivation for 24 h or 48 h, as previously described.^62^ Control mice had *ad libitum* access to food. Blood samples were collected from the tail tip at baseline, after 24 h, and after 48 h in the case of mice subjected to 48 h of STS. Liver and fat pad samples were harvested at the corresponding time points following euthanasia. Mice weight was measured every day. All mice had unlimited access to drinking water at all times. For all mouse experiments, group sizes were based on expected differences and numbers of available mice. No exclusion criteria were set for measurements of body weight, serum proteins, or blood glucose levels, and no datapoints were excluded from the analysis, except for three serum insulin measurements from Akita mice in which excessive hemolysis occurred. We found consistent results in male and female mice wherever both sexes were assessed.

#### Ethics

All animal experiments were conducted in accordance with the protocols approved by the institutional animal care committees at Lund/Malmö (permit number 20069/2020 and 22112/2025), or at Universidad de Navarra (permit number 131-22), where the procedures were carried out.

### Method details

#### Blood glucose measurements

These were taken using an Accu-Chek Aviva blood glucose meter, on blood samples from the saphenous vein. Serum samples were also taken from the saphenous vein in a manner preserving complement activity: collected blood was allowed to rest at room temperature for 20 minutes, then on ice for 15 minutes, before spinning at 7500 G for 10 minutes at 4°C, and serum aliquots frozen directly and stored at -80°C. For fasting blood glucose and insulin levels, food was removed from cages for 4 h before blood/serum collection.

Insulin treatment of diabetic animals: In STZ-induced diabetic mice, insulin-containing 3 mm implants or mock “blank” implants (LinShin Canada Inc) were implanted subcutaneously under dorsal skin by injection through a trocar, according to supplier instructions.

qPCR: Mice were euthanized by cervical dislocation and tissue samples were immediately collected by dissection, flash-frozen in liquid nitrogen, and stored at -80°C. For RNA extraction, samples were ground in individual RNAse-free mortar and pestle tubes (Fisher Scientific), or homogenized in an H24 tissue homogenizer (TianGen) using 5 mm steel beads. RNA was then immediately extracted using QIAzol / chloroform extraction and RNEasy mini-columns (QIAGEN). RNA quality was determined by measuring 260/280 and 260/230 nm absorbance ratios on a Biodrop spectrophotometer, and integrity was assessed by RNA ScreenTape analysis (Agilent). Samples were excluded if RIN values were below 0.7. Samples from at least 4 mice were included in all experiments. For STS mice, 4 tissue samples were chosen blind per group to analyse by qPCR. For Akita mice, tissues from 4 Akita and 4 age-matched WT littermates were taken from each group for RNA extraction and qPCR analysis. Samples from the mid-range of the age range of the cohort were chosen. Reverse transcription was performed using Superscript IV transcriptase (ThermoFisher) and qPCR was performed using Taqman qPCR assays (ThermoFisher) on a Viia7 Real-Time PCR system (ThermoFisher). Expression values were calculated using the ΔCt method, using geometric means of expression to reference genes beta-2 microglobulin and HPRT, except panels 1A, 1B, where ΔΔCt method was used, with the same reference genes. The qPCR operator was blinded to sample identity, which was revealed after analysis. No datapoints were excluded from the analysis.

AP activity assays: AP was assessed by deposition of C3 onto zymosan particles, measured by flow cytometry. Serum aliquots were thawed on ice and diluted to 10% in AP assay buffer (PBS with 5 mM MgCl_2_ and 10 mM EGTA), which prevents classical and lectin pathway activation. EDTA (20 mM) was added to negative control samples. Zymosan beads were added and incubated at 37°C for 20 minutes, shaking at 300 rpm. Zymosan beads were then washed 2x in ice-cold PBS with 1% BSA and resuspended in 1:100 FITC-labeled goat anti-mouse C3 antibody (ICN, #55500). After 1 h incubation on ice, samples were washed 2x in PBS 1%BSA and run on a CytoFlex flow cytometer (Beckman Coulter). Zymosan particles were gated by forward and side scatter, and gMFI values were collected.

ELISA: FB FD and C3 were measured in mouse serum samples using a mouse factor B ELISA kit (Novus Biologicals #NBP2-75243), a mouse factor D/adipsin DuoSet (R&D systems, #DY5430) and a mouse C3 ELISA kit (Abcam #ab263884), respectively. Serum insulin was measured using an ultrasensitive mouse insulin ELISA (Mercodia #10-1249) and mouse C3b was measured in serum samples using an ELISA kit from Hycult Biotech (HK216). ELISA operator was blinded to the sample identities until after analysis of values.

Western blotting: Serum samples were diluted in PBS and boiled after the addition of Laemmli buffer containing 50mM DTT, before running on SDS-PAGE. Proteins were transferred to PVDF membranes, blocked for 1 h in blocking buffer (10% fish gelatin (Norland Products) diluted in TBS with 0.1% Tween-20). The following primary antibodies were then added and incubated at 4°C overnight, diluted 1:1000 in blocking buffer: rabbit anti-C3 (Abcam #ab200999), sheep anti-mouse FD (R&D systems, #AF5430), goat anti-CFB (Complement Technologies #A235). Membranes were then washed 3 times for 5 minutes in TBS-tween, before incubating 1 h with HRP-labeled anti-rabbit/sheep/goat secondary antibodies as appropriate (DAKO) diluted 1:1000 in blocking buffer. Membranes were then washed 3 times in TBS-Tween before developing using ECL substrate (Millipore) and images captured by ChemiDoc MP imaging system (BioRad). For blotting tissue homogenates, approximately 50 mg of tissue was placed in a 2 ml tube with 250 ul of RIPA buffer containing 1% SDS, and homogenised in a H24 tissue homogeniser (TianGen) using 5 mm steel beads. Samples were cleared by centrifugation, and then BCA assay (Pierce) used to quantify total protein content. 5 ug was loaded per lane for Western blot.

External data: Microarray data from low-fat diet (LFD) and high-fat diet (HFD)-fed male mouse epididymal fat pads (GEO: GSE123394) were analysed at https://www.ncbi.nlm.nih.gov/geo/geo2r/?acc=GSE123394, and were generated as described in reference.^29^ Array data from male C57Bl/6 lean and ob/ob mice were downloaded from the Attie lab diabetes database (http://diabetes.wisc.edu/) and were generated as described.^31^ Array data (GEO: GSE39752) from livers of STZ-treated mice and controls as described in reference^34^ were analysed at https://www.ncbi.nlm.nih.gov/geo/query/acc.cgi?acc=GSE39752.

### Quantification and statistical analysis

Statistics were calculated using GraphPad Prism. The experimental unit was one animal. Graphs plot average values per animal, and mean and standard deviations for each group. Statistical tests used included t-test for comparing two groups, one-way ANOVA for comparing more than two groups, or two-way ANOVA for comparison of multiple variables, with Bonferroni post-tests for multiple corrections. Correlations show the line of best fit with 95% confidence intervals. In all graphs, ∗, *p* < 0.05, ∗∗, *p* < 0.01, ∗∗∗, *p* < 0.001, ∗∗∗∗, *p* < 0.0001, as shown in figure panels. Descriptions of statistical analyses can also be found in figure legends. Raw data used in this manuscript is available from the authors on request.
