# Supplementary material for: Type 2 and type 1 diabetes have opposing effects on the systemic murine complement alternative pathway
Source: iScience. 2026 Jun 12;29(7):116359. doi: 10.1016/j.isci.2026.116359 (PMC13276781; doi:10.1016/j.isci.2026.116359)
Supplement: Document S1. Figures S1 and S2 [file mmc1.pdf]

iScience, Volume *29*

Supplemental information

Type 2 and type 1 diabetes have opposing effects on the systemic murine complement alternative pathway

Lucie Colineau, Olga Kolodziej, Daniel Ajona, Ruben Pio, Anna M. Blom, and Ben C. King

Supplementary figure 1, for figure 1

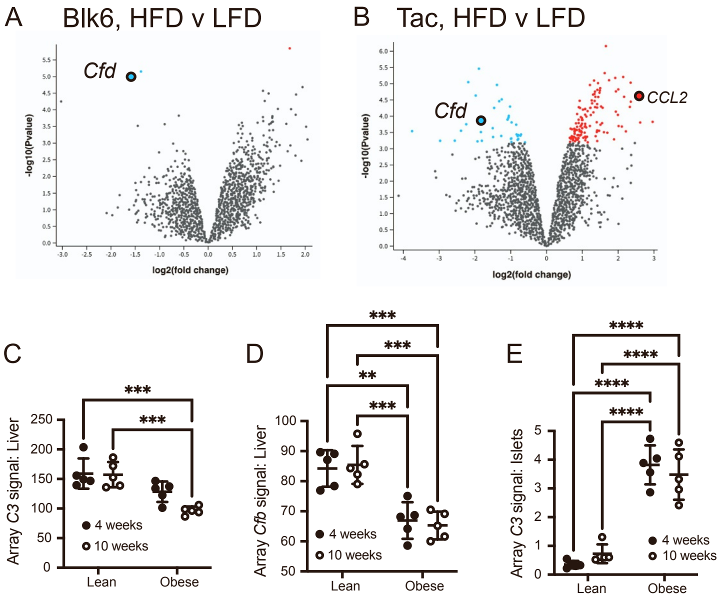

Supplementary figure 2, for figure 2:

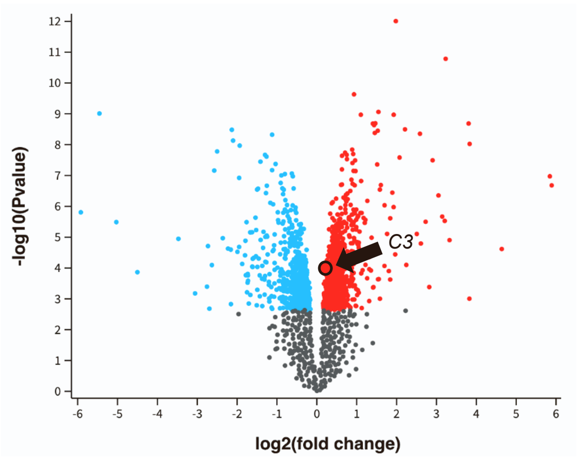

Supplementary figure legends:

Supplementary Figure 1: Corroborating publicly available data from diet-induced obesity models: A) Volcano plot of differentially expressed genes in epididymal adipose tissue determined by microarray from HFD- versus LFD-fed mice (n = 4 per group) on the C57Bl/6 background, showing *Cfd* (highlighted) as one of the most strongly and significantly downregulated genes in HFD mice. B) Similar data from HFD versus LFD mice on the Tac genetic background (n = 3 per group). C) RNA microarray signal for *C3* from livers of lean or ob/ob mice on the C57Bl/6 background at different ages. D) As (C), but showing array values for liver *Cfb* expression. E): As (C), but showing *C3* expression values in pancreatic islets, demonstrating C3 upregulation. See methods for descriptions of data sources. In volcano plots, significance cutoff was set at Padj < 0.05. For panels C-E, **, $p < 0.01$, ***, $p < 0.001$, ****, $p < 0.0001$, by 2-way ANOVA.

Supplementary figure 2: RNA microarray data from livers of T1D model STZ-treated (n = 6) versus untreated mice (n = 7), showing a significant upregulation of *C3* expression (highlighted) in diabetic mice. See methods for data source. Significance cutoff was set at Padj < 0.05.
